# Research progress on complications of rheumatoid arthritis

**DOI:** 10.3389/fimmu.2025.1561926

**Published:** 2025-05-27

**Authors:** Haiqing Zeng, Zedongfang Yuan, Run-tian Wu, Zhisheng Huang

**Affiliations:** ^1^ Guangzhou University of Chinese Medicine, Guangzhou, China; ^2^ Guangzhou Hospital of Integrated Traditional and Western Medicine, Guangzhou, China

**Keywords:** complications, research progress, rheumatism, autoimmune disease, rheumatoid arthritis

## Abstract

Rheumatoid arthritis (RA) is an autoimmune disease characterized by synovial inflammation, joint injury, and deformity in the limbs. This disease is widely distributed, with a large number of patients, and there is a possibility of further increasing the number of patients in the current aging society. RA can cause complications in multiple systems, many of which can seriously affect patients’ quality of life and even significantly increase the risk of death. The unclear mechanism of RA makes it difficult to achieve accurate prediction and treatment of its complications. Therefore, this article provides an overview of the research progress on the common or extensively studied complications of RA, such as joint injury, interstitial lung disease, vasculitis, osteoporosis, Sjogren's syndrome, malignant tumors, etc. It identifies the problems and shortcomings in research and proposes some suggestions for future measures, in order to raise awareness among medical professionals and the public about actively controlling these complications and provide some assistance.

## Introduction

Rheumatoid arthritis is an autoimmune disease based on synovitis whose mechanism has not yet been identified. This disease not only has the characteristic symptoms of joint pain and swelling, but also can further lead to a variety of extra articular diseases (1).At present, the treatment of rheumatoid arthritis is still mainly to control the disease progression, and there are still a large number of patients with the disease whose condition is difficult to be effectively controlled (2), so most patients will suffer from the disease for life. In North America and Western Europe, the prevalence of rheumatoid arthritis can reach 0.5% to 1%, and studies have shown that the prevalence of this disease can increase with age (3), so with the progress of the aging of the world population, the risk of suffering from this disease will continue to increase. It is obvious that, similar to diabetes, actively controlling the complications of rheumatoid arthritis can effectively reduce the social burden of the disease. However, most of the current studies on the complications of rheumatoid arthritis only focus on a single complication and lack systematic understanding. Therefore, this review summarizes the research on the complications of the disease so far to provide some reference for relevant researchers.

## Joint damage

As a disease based on synovial inflammation, the most direct complication of rheumatoid arthritis is joint damage caused by persistent inflammation. Studies have shown that 39%-73% of patients with early rheumatoid arthritis have joint erosion of the hands within 5 years. In addition (4), joint destruction has been considered as one of its characteristics (5), which shows the high probability of this complication in rheumatoid arthritis. At present, the mechanism of joint destruction in rheumatoid arthritis is not clear, but research has found that synovial mesenchymal cells driven by transcription factor c-Fos/AP-1 (6), fibroblast like synovial cells (7), osteoclasts, cytokines including TNF, IL1, IL17, rheumatoid factors, anti CCP antibodies and other autoantibodies all play a role in the occurrence of joint destruction in rheumatoid arthritis (8). Specific to the clinical manifestations, the joint destruction caused by rheumatoid arthritis can be manifested as joint swelling, pain and tenderness (9), bone destruction such as periarticular bone erosion, cartilage degradation as well as joint destruction commonly seen in hands and feet,etc. (10, 11) In terms of treatment, in addition to the basic treatment of rheumatoid arthritis such as oral Disease-modifying anti-rheumatic drugs(DMARDs) and non steroidal anti-inflammatory drugs(NSAIDs), there are also some more specific research on the treatment of rheumatoid arthritis joint destruction, such as denosumab and rituximab, which have obvious inhibitory effect on RA joint destruction (12, 13); TNF blockers that have a profound and sustained inhibitory effect on RA joint bone erosion (14); Histone-modifying inhibitors can remodel RA synovial fibroblasts cells that promote osteoclast bone erosion to inhibit joint bone destruction (15); Small molecule inhibitor of cathepsin K that can directly inhibit the absorption of osteoclasts (16), and joint protection surgery that can effectively alleviate joint destruction (17). In terms of future treatment prospects, ideas such as reducing bone destruction by regulating the metabolic pathway of osteoclasts have also been proposed (15), but it is still important to clarify the best treatment method with sufficient evidence and the treatment with better effect.

## Interstitial lung disease

Lung disease has been recognized as one of the important extra articular complications of rheumatoid arthritis (18).Although the complications of RA in the lung are diverse, interstitial lung disease has a high prevalence and is often one of the main complications that lead to the death of RA patients (19).Specific to the number, some studies believe that the prevalence of RA complicated with interstitial lung disease is 3.6% (20), while others believe that the prevalence is between 3.7% and 7.7%, or even higher (21). The differences may be due to the regional and ethnic differences of the experimental population, as well as the inevitable deviation in the collection of patient information. However, these findings vividly prove the high risk of this complication and deserve the attention of medical workers. At present, the clear etiology and pathogenesis of rheumatoid arthritis related interstitial lung disease are not yet clear. However, according to reports, some factors such as ACPA, gene mutations, smoking, environment, autoimmunity and other factors are involved in the pathogenesis of rheumatoid arthritis associated interstitial lung disease (19, 22),and these factors lead to abnormal tissue reaction between alveolar wall and lung parenchyma (19).Some studies have interpreted the mechanism of lung involvement as the damage of airway and alveolar epithelial cells leading to the activation of immune cells and the excessive accumulation of extracellular matrix in lung tissue cells, but the current evidence is still difficult to fully define it (23).In terms of clinical manifestations, rheumatoid arthritis related interstitial lung disease can often have the manifestations of exertional dyspnea and dry cough in the early stage (24). Shortness of breath and bibasilar inspiratory crackles are also considered to be common (25, 26). In the late stage of this complication, clubbing often occurs (27). However, it should be noted that these symptoms are also common in other respiratory diseases. Clinical attention should be paid to the identification to minimize misdiagnosis and omissions. In the diagnosis of this complication, on the basis of the above symptoms, combined with higher inflammatory activity, higher ACPA value, MCP-1/CCL2, IL-18, SDF-1 and other inflammatory factor levels (28), Pulmonary Function Tests(PFTs), bronchoalveolar lavage, HRCT examination (29), lung ultrasound(LUS), serum KL-6 antigen level (30), usually can achieve a more accurate diagnosis, but whether patients need to do all the above-mentioned auxiliary examinations in order to make a clear diagnosis is still unclear. It may be a better choice to consider the clinical and patients’ comprehensive situation. In terms of treatment, the situation is more complex. On the one hand, immunosuppressants are often used as the first choice for rheumatoid arthritis complicated with interstitial lung disease (31); On the other hand, the use of immunosuppressive agents such as methotrexate is limited because the disease is common in the elderly (32), so the use of other drugs should also be paid attention to. Compared with TNF inhibitors, studies have shown that non TNF inhibitors such as abatacept, tocilizumab and rituximab have better efficacy in the treatment of this disease (33). However, antifibrotic drugs, such as nintedanib, have shown good effects in preventing and delaying the progression of the disease, and can be used as adjuvant drugs (34). In the acute exacerbation of the disease, no targeted treatment has yet been produced. At present, high-dose glucocorticoids combined with immunosuppressants are still used to control the disease (35), and a better treatment scheme remains to be studied. If the above drug regimens fail to effectively control the disease, there are also studies showing that lung transplantation can improve dyspnea and quality of life in changing play (36).

## Vasculitis

Rheumatoid vasculitis(RV) is a complication that directly affects the blood vessels of patients with rheumatoid arthritis and causes vascular inflammation. It can occur in blood vessels of almost all sizes, and it is a rare complication of rheumatoid arthritis with poor prognosis and high mortality (37). Although new treatments with good curative effects such as biotherapy have reduced the incidence of RV to less than 3.9 per million people today (38), the highest five-year mortality rate of more than 60% suggests that we should not ignore this disease (39). The pathogenesis of this complication is not completely clear, but many studies have shown the important role of genetic factors in this mechanism (40–42), and some studies have suggested that the formation of immune complexes promotes the recruitment of neutrophils (43), endothelial cell activation, inflammatory factor production,etc. (44), and then produces vascular inflammation, leading to the damage of vascular wall. In terms of specific symptoms, the skin manifestations of RV are relatively common (45). If it is mainly small vessel inflammation, it can often cause skin infarct papules of fingers, as well as skin purpura, congestion, maculopapular rash; If inflammation occurs in larger blood vessels, it can cause nodules, racemose moss, necrotic ulcers, etc. (45) Vasculitis can also cause peripheral nerve infarction, causing neuritis, distal symmetric sensory or motor neuropathy, in which the lower limbs are more prone to occur than the upper limbs (46); The heart can also have pericarditis, tamponade and other critical symptoms due to RV (39); In the eyes, RV can cause scleritis, keratitis, etc (47, 48); In addition, RV can also cause fatigue, fever, weight loss, muscle pain and other difficult to identify symptoms (49).At present, when diagnosing RV, one of the above clinical manifestations and skin or muscle vasculitis related tissue biopsy is often selected (50), but because the biopsy may be false negative, there are also studies suggesting that the combination of serum rheumatoid factor IgA and C3 complement test results can be considered to assist the diagnosis (51), which also enlightens that the current diagnostic methods still need to be improved. For the moment, there is no unified standard for the treatment of RV (39). Glucocorticoids, DMARDs, cyclophosphamide, cyclosporine, biological agents and other drugs are used in the treatment of the disease (52, 53). Tnfi and rituximab are used as the first-line drugs for the disease because of their good remission effect (54–56), but the formulation of more standardized treatment and management standards is still very important.

## Osteoporosis

Rheumatoid arthritis is also an important factor that causes bone mineral density reduction, osteoporosis, and further fractures (57).Studies have shown that compared with the general population, the probability of osteoporosis in patients with rheumatoid arthritis is significantly increased by about 2 times (58). If patients with RA do not receive glucocorticoid treatment, the incidence rate can reach 7.5 per 1000 people per year (59), and the prevalence rate can reach 30% (60). If women with RA are postmenopausal, about one-third of the population can suffer from osteoporosis (58). Considering the significantly elevated fracture risk of patients with osteoporosis, it is urgent to control this complication. In terms of pathogenesis, studies have shown that RA leads to osteoporosis mainly through its effects on osteoclasts and osteoblasts (61). RA can promote the formation of osteoclasts by affecting RANKL, T cells, B cells, etc. (62, 63), while abnormally formed osteoclasts can cause bone resorption and erosion by releasing catalytic decomposition enzymes and acid enzymes (64). On the other hand, RA can also affect the normal formation and development of osteoblasts by affecting bone morphogenetic protein (BMP), resulting in insufficient sources of bone formation (65). In terms of clinical manifestations, as a relatively hidden disease, RA related osteoporosis may not show obvious symptoms in the early stage, so the study of symptoms has not attracted enough attention. However, with the development of the disease, fatigue, pain (66), spinal deformation (67), and fracture may occur in severe cases (68), which often makes the disease get due attention at this time. In terms of diagnosis, a unified standard for the diagnosis of this complication has not yet been formulated, but studies have suggested that combining the gold standard examination of osteoporosis - bone mineral density examination with serum 25 hydroxyvitamin D and bone turnover marker β-CROSSL level examination can accurately predict the occurrence and development of the disease (69), which is worthy of our reference, but more relevant studies are still needed to confirm and enrich the diagnostic methods. In terms of treatment, the current research basically believes that the treatment of this complication is achieved through the basic medication of RA combined with anti osteoporosis drugs (70–72), but the specific usage will be different. Glucocorticoids have the risk of aggravating the progression of osteoporosis, so we should pay attention to control the dosage of glucocorticoids and actively use anti osteoporosis drugs at the same time (70). A variety of biological agents have shown protective effects on bone mass while inhibiting RA inflammation, such as Tofacitinib and Iguratimod (73). However, currently, anti osteoporosis drugs represented by bisphosphonates are still used as first-line drugs, because they can show better effects (70). In addition, non drug treatment has also attracted attention. Adequate intake of calcium and vitamin D, smoking cessation, reduction of alcohol consumption, assessment of fall risk, maintenance of normal weight, avoidance of bad living habits such as sitting for a long time, and active physical exercise are all recommended to patients with RA complicated with osteoporosis to better ensure the curative effect (70, 71). However, the current scheme is still not the best, and the role of treatment is still biased towards maintenance rather than cure (71, 72). Therefore, a better treatment scheme for this complication still needs our efforts.

## Sjogren’s syndrome

Rheumatoid arthritis can also cause the same type of disease, Sjogren’s syndrome is one of them (74).In patients with RA, this complication can occur in about 6% to 9% of patients (75). Studies have shown that secondary Sjogren’s syndrome will worsen the condition and increase the mortality of RA patients (76). At present, the pathogenesis of this complication is not clear, but studies have found that as many as 322 genes are commonly associated with RA and Sjogren’s syndrome, among which CXCL10, GZMA, IGTA4, PSMB9 and other genes are closely related to the activity of these two diseases (77), and megakaryocytes may also play an important role in the occurrence of these two diseases (78). Although there is still a lack of research on the mechanism of directly linking the two diseases, the above research may also suggest that the two are linked through gene and cell pathways. Of course, this still needs further research and demonstration. In terms of clinical manifestations, the manifestations of Sjogren’s syndrome secondary to RA are not significantly different from those of primary Sjogren’s syndrome. They are dry eyes caused by lymphoid infiltration of exocrine glands and epithelial cells involving lacrimal and salivary glands and salivary dysfunction with dry mouth as a common manifestation (79), but secondary Sjogren’s syndrome will also show symptoms of RA.In terms of diagnosis, the diagnostic criteria for Sjogren’s syndrome secondary to RA have not yet been formulated, so at present, the classification criteria for primary Sjogren’s syndrome formulated by the American College of Rheumatology/European League Against Rheumatism in 2016 are still mostly referred to (80), such as lymphocytic lip salivary gland inflammation, anti ssa/ro positivity, higher eye stain score, Schirmer test, unstimulated salivary flow, combined with the above symptoms of dry eyes, dry mouth, and earlier diagnosis of RA (81). In addition, some studies have also found the good effect of serum chemokine CCL28 in the diagnosis and evaluation of Sjogren’s syndrome (82), which also suggests that the methods for the diagnosis of this disease still have development potential. In terms of treatment, studies have suggested that abatacept has a good effect on improving the glandular and extraglandular involvement, systemic disease activity and patient reported outcomes in patients with RA related Sjogren’s syndrome, but more high-quality RCTs are still needed to support it (83); Some studies have also found that human chorionic gonadotropin has shown good remission effect on RA and Sjogren’s syndrome, which can be used as an adjuvant treatment option (84). However, in general, the research on the treatment of RA related Sjogren’s syndrome is insufficient, so the current treatment plan for a separate Sjogren’s syndrome is also worthy of reference. The stability of lysosomes found in relevant studies, interferons such as MHV370, RSLV-132, CAR-T cell therapy, epalizumab, seletalisib and other therapies have shown good efficacy and therapeutic potential for Sjogren’s syndrome. What is more worthy of expectation is that a large number of drugs for the treatment of Sjogren’s syndrome are under clinical research at different stages (85), which may suggest that the formulation of standard treatment regimens for this complication will benefit in the near future.

## Malignant tumor

Rheumatoid arthritis has also been recognized as a risk factor for a variety of malignancies (86). Malignant tumor, as we all know, is a disease with poor prognosis, which is still one of the main diseases leading to death until now (87). Many studies have suggested that RA can increase the risk of malignant tumors. Although the specific data are still unclear, the increase can reach 50% (88, 89), which makes us not ignore the existence of RA in the occurrence and treatment of malignant tumors. In the pathogenesis, some studies have shown that the mechanism of RA leading to malignant tumors may be due to long-term chronic inflammatory stimuli (90). Some studies also believe that common triggers such as smoking and airway inflammation may induce RA and malignant tumors at the same time (91–94). On the other hand, genetic and environmental factors have also attracted some attention (95), but in general, these mechanisms are not clear enough and need more experimental proof.In terms of symptoms and diagnosis, there is not enough research to explain the specific symptoms and diagnostic characteristics of RA related malignant tumors. Interleukin-6 is considered a potential biomarker linking rheumatoid arthritis and malignant tumors, with the potential to monitor RA associated malignant tumors, but further experimental evidence is needed (96).Time has been considered by some studies as the method to determine the complications (89), but there are inevitably many interfering factors, making time not the best diagnostic method. Therefore, enough attention and a large number of experiments are needed to support the research on the special symptoms and diagnostic basis of this complication. In terms of treatment, there is no unified and objective guidance plan for the treatment of RA related malignant tumors, but some good suggestions can still be found from the existing expert consensus. Compared to biological Disease-Modifying Anti-Rheumatic Drugs(bDMARDs),conventional synthetic Disease-Modifying Antirheumatic Drugs(csDMARDs) are considered safer for active cancer. Compared to targeted synthetic Disease-Modifying Anti-Rheumatic Drugs(tsDMARDs), conventional synthetic Disease-Modifying Antirheumatic Drugs are also considered a more reliable choice. However, at the same time, individual factors such as the patient’s age, cancer type, stage, prognosis, and values should also be considered when choosing medication (95); The use of corticosteroids is controversial as they may have both positive and negative effects on cancer, and therefore should be used with caution (97, 98); If the patient’s cancer requires surgical treatment, non steroidal anti-inflammatory drugs, immunosuppressants, and other medications used to treat RA may be discontinued to prevent risks such as bleeding and infection (99, 100). In terms of radiation therapy, no significant effect of radiation therapy on RA has been found yet, but caution should still be exercised when using it (95). When facing chemotherapy, targeted drug therapy, immune checkpoint inhibitors, and other treatments, attention should be paid to the interaction with anti rheumatic drugs and the possible adverse consequences (95, 101). In summary, RA related malignant tumors can usually be treated with both RA and malignant tumors simultaneously, but monitoring of efficacy, adverse reactions, and more comprehensive and universal treatment guidelines are still needed.

## Cardiovascular diseases

Given that vasculitis has been taken into consideration, the risk of cardiovascular disease cannot be ignored. From an epidemiological perspective, RA can increase the probability of cardiovascular disease in the human body by 48% and increase the mortality rate related to cardiovascular disease by 50%. Among RA patients, 4.68% may experience cardiovascular outcomes. Cardiovascular disease accounts for the largest proportion of excess mortality in RA, at approximately 39.6% (102, 103). In terms of pathogenesis, current research suggests that the cardiovascular disease outcome of rheumatoid arthritis is mainly caused by inflammation. Rheumatoid arthritis leads to the abundant presence of inflammatory factors such as interleukin(IL)-1,IL-6 and tumor necrosis factor (TNF) in the systemic circulation. These inflammatory factors can cause vascular endothelial dysfunction, and affect lipid metabolism, causing abnormal activity and expression of lipoprotein esterase, reducing high-density lipoprotein cholesterol, increasing low-density lipoprotein cholesterol, decreasing levels of apolipoprotein A1 and increasing levels of apolipoprotein B. Disruptive lipid metabolism can further promote inflammatory reactions and increase blood viscosity, and then lead to atherosclerosis and more serious cardiovascular diseases. At the same time, the increased secretion of proteolytic enzymes and the expression of interferon - γ (IFN - γ) in rheumatoid arthritis also promote plaque instability, which increases the risk of severe cardiovascular disease (104).The clinical manifestations of rheumatoid arthritis can include pericarditis, cardiomyopathy, arrhythmia, atherosclerosis, heart failure, valvular heart disease, of which the most common is considered to be ischemic heart disease represented by atherosclerosis and congestive heart failure (105, 106).In terms of diagnosis, current research mainly focuses on predicting and early detecting cardiovascular complications in patients with rheumatoid arthritis, including monitoring blood lipids, arterial ultrasound examination, cardiovascular magnetic resonance imaging, and other methods to detect cardiovascular lesions as early as possible (107–109).In addition, biomarkers such serological asasymmetric dimethylarginine(ADMA),interleukin-17A and osteoprotegerin that have the potential to predict cardiovascular complications in patients with rheumatoid arthritis are also being studied (110, 111).In terms of treatment, current research still focuses on controlling the inflammation and disease activity of rheumatoid arthritis to reduce the probability of cardiovascular complications or alleviate existing cardiovascular complications. The main therapeutic drugs are still traditional nonsteroidal anti-inflammatory drugs, glucocorticoids, conventional synthetic anti rheumatic drugs, biologics, targeted synthetic anti rheumatic drugs (104).However, some studies have also found the potential of JAK inhibitors in the treatment of rheumatoid arthritis combined with cardiovascular disease, which may indicate the emergence of new and more targeted therapeutic drugs in the near future (112).

## Advanced therapy

Advanced therapy refers to a new type of treatment that utilizes cutting-edge biological technologies such as cells and genes, which is a new choice for current and future difficult diseases. At present, the mechanism and treatment research of rheumatoid arthritis is not sufficient, and the current drugs for treating rheumatoid arthritis are still not perfect. Therefore, advanced treatment may be a promising method. Due to the lack of targeted strategies for the treatment of complications of rheumatoid arthritis, most of them still focus on treating the inflammation itself. Therefore, the current advanced treatment for its complications is also based on the study of treating rheumatoid arthritis itself.

MicroRNAs are a class of non coding single stranded DNA molecules that exist in eukaryotes and are involved in post transcriptional regulation of gene expression. When their regulatory ability is disrupted, immune inflammations including RA will further develop (113).Based on this mechanism, researchers currently studying interventions targeting microRNAs such as hsa-miR-21-5p and miR-340-5p that are highly associated with rheumatoid arthritis to control the condition of rheumatoid arthritis (114).Perhaps we will soon see the emergence of specific drugs and their expected effects.

Genome editing technology is a cutting-edge technology that precisely modifies specific target genes in the genome of organisms. The Clustered Regularly Interspaced Short Palindromic Repeats(CRISPR)-Cas9 system is a genome editing technology developed based on bacterial natural immune systems that can accurately identify and cleave DNA sequences, its precise characteristics make it a good tool for gene editing therapy for rheumatoid arthritis (115).Another condition that makes gene editing technology possible for treating rheumatoid arthritis is the numerous genetic related studies that have been completed, such as large-scale genome wide association studies (GWAS).The genes IL-20RA, OLIG3, miR-155, and others discovered in current research are considered suitable choices for treating rheumatoid arthritis through CRISPR-Cas9 technology (116).However, considering the off target risks and ethical concerns that still exist in the technology, the specific application of treatment may take some time.

Mesenchymal stem cell (MSC) therapy is a cutting-edge therapy that utilizes the self-renewal and differentiation abilities of mesenchymal stem cells to treat diseases. Due to the fact that mesenchymal stem cells can participate in regulating the activity of the immune system through various pathways to achieve inhibitory effects on inflammatory responses, these cells have certain potential for controlling the condition of rheumatoid arthritis (117).Some completed studies on mesenchymal stem cell therapy for rheumatoid arthritis have shown that this treatment is effective and relatively safe for rheumatoid arthritis, but the current high cost of this treatment makes it not yet recommended as one of the top priority treatments. When mesenchymal stem cell therapy becomes an alternative treatment for traditional treatments such as DMARD or biologics, the greater risks of infection, thrombosis, tumors, etc. will also plague the choice of treatment, and these issues are urgent to be addressed (118).But overall, mesenchymal stem cells are a promising approach for treating rheumatoid arthritis, and improving medication methods and reducing treatment costs will promote further promotion of this method.

Adoptive regulatory T cell therapy is a treatment method that involves modifying regulatory T cells extracted from healthy individuals or patients *in vitro* and reintroducing them into the patient’s body to enhance the immune system’s regulatory function. Regulatory T cells(Tregs) refer to a subset of T cells that can control the immune response in the human body, and have inhibitory and anti-inflammatory effects on the body’s immune response. Existing research suggests that this therapy has a significant inhibitory effect on inflammation, but currently there are not many studies on this treatment for rheumatoid arthritis, and the risks of regulatory T cells being converted into pathogenic cells and easily contaminated during extraction still need to be addressed (119). Therefore, more relevant experiments are still needed to further confirm the effectiveness and safety of this treatment for rheumatoid arthritis.

Chimeric antigen receptor(CAR) T-cell therapy is a cutting-edge treatment method that has recently received a lot of attention. Similar to the adoptive regulatory T-cell therapy mentioned above, its method is to modify and extract T cells from patients’ bodies *in vitro*, making them more powerful in treating specific pathogens. This therapy was initially researched around treating tumors, but as some researchers attempted to use it to treat immune diseases such as systemic sclerosis and achieved good results, researchers believe that it may also have great potential for rheumatoid arthritis (120).Given the complex characteristics of rheumatoid arthritis, current research on CAR-T cell therapy for the treatment of rheumatoid arthritis includes precise treatment through various methods, such as labeling rheumatoid arthritis specific serum markers with fluorescein isothiocyanate (FITC), rheumatoid arthritis specific associated HLA DR alleles and their self antigen peptides, and using chimeric autoantibody receptor T cells (CAAR-T) and chimeric antigen receptors in regulatory T cells (CAR Tregs), but the most suitable method has not been determined yet, and more experiments are still needed to confirm the efficacy and avoid risks (121–123).CAR-T cell therapy is considered a highly promising treatment for rheumatoid arthritis, but its promotion and application are still greatly limited until the risks of severe inflammation and high costs are resolved.

Overall, there is a wealth of research on advanced treatment methods for rheumatoid arthritis, but there is generally a lack of sufficient clinical trials to prove their efficacy and safety. If considering the treatment of complications of rheumatoid arthritis, the specificity of these treatments is still not precise enough. However, as therapies that are very different from traditional treatments for rheumatoid arthritis and its complications, we have reason to believe that they may replace existing therapies in the future to better control the condition of rheumatoid arthritis and its complications with higher efficacy and safety.

## Summary

**Figure 1:**
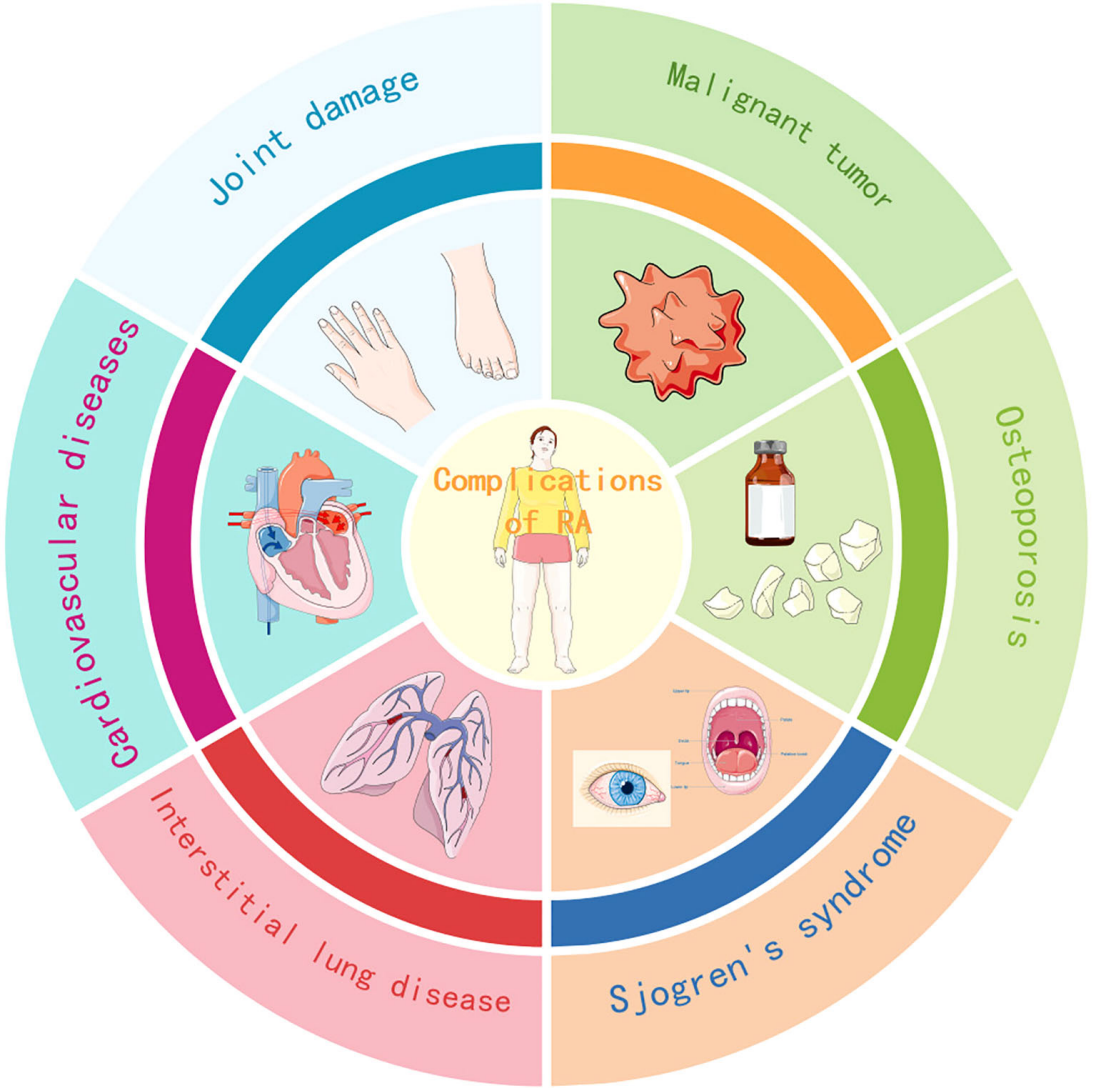
Common and highly concerned complications of rheumatoid arthritis. This study mainly discusses the complications of six common and widely studied types of rheumatoid arthritis, including joint injury, interstitial lung disease, osteoporosis, Sjogren’s syndrome, cardiovascular disease (including vasculitis), and malignant tumors.

As a disease whose mechanism cannot be elucidated yet, rheumatoid arthritis can not only affect local joints, but also multiple human systems or organs such as the respiratory system, cardiovascular system, and bones, and may induce other autoimmune diseases, malignant tumors, etc., as shown in **Figure 1**. These complications increase the risk of life-threatening situations, which warns us not to ignore further understanding and active treatment of rheumatoid arthritis complications. Unfortunately, although this disease has been discovered for over a hundred years, the mechanism, characteristics, and treatment of its complications have not been fully understood, which is a significant obstacle to achieving the goal of improving disease prognosis. However, at the same time, understanding the complex mechanisms of rheumatoid arthritis may require more time and manpower to determine the characteristics of its complications, as well as diagnosis and treatment. Although the diagnosis and treatment guidelines for complications of rheumatoid arthritis have not been formulated yet, these diseases are gradually attracting more and more attention and research. It is also evident that rheumatoid arthritis patients have greatly reduced the risk of deterioration due to the use of anti rheumatic drugs, the advanced treatment for it has also shown good results and has great potential. Therefore, we should still believe that a bright future is not far away.
